# Intraperitoneal doxorubicin achieves access to mesenteric lymph nodes, case series of two patients^☆^

**DOI:** 10.1016/j.ijscr.2018.11.065

**Published:** 2018-12-12

**Authors:** Paul H. Sugarbaker, O. Anthony Stuart

**Affiliations:** Program in Peritoneal Surface Oncology, Washington Cancer Institute, Washington, DC, United States

**Keywords:** Intraperitoneal chemotherapy, Doxorubicin, Mesenteric lymph nodes, Pharmacokinetic studies, Hyperthermic intraperitoneal chemotherapy (HIPEC), Preperitoneal lymphatic network, Intestinal adhesions, Peritoneal metastases, Case series

## Abstract

**Background:**

Intraperitoneal chemotherapy has been used extensively to treat cancers that metastasize to the peritoneal surfaces. In the operating room the intraperitoneal chemotherapy is administered with moderate heat. The chemotherapy is used after surgery when there is minimal residual disease. Alternatively, intraperitoneal chemotherapy can be used long-term in an attempt to prevent the progression of disease in patients at high risk for peritoneal metastases to occur during follow-up.

**Methods:**

Hyperthermic intraperitoneal chemotherapy (HIPEC) with doxorubicin was used to treat peritoneal metastases in two patients. These two patients required generous sampling of mesenteric lymph nodes because involvement of these lymph nodes was suspected on preoperative radiologic studies by computed tomography (CT). The lymph nodes, peritoneal fluid and plasma along with tumor tissue in one patient were subjected to a determination of doxorubicin chemotherapy concentrations by high pressure liquid chromatography (HPLC).

**Results:**

The ratio of doxorubicin present in peritoneal fluid as compared to plasma was 80 in patient 1 and 50 in patient 2. There was an increased doxorubicin concentration within lymph nodes as compared to plasma. The accumulation of doxorubicin in lymph nodal tissue was 40 times that of plasma in patient 1 and 30 times that in patient 2. In patient 2, the concentration of doxorubicin in lymph nodes was very similar to the concentration of chemotherapy within tumor nodules.

**Conclusion:**

Doxorubicin chemotherapy was not only present in peritoneal surface tissue but also a surprisingly high level was present in mesenteric lymph nodes in patients treated by HIPEC. This observation may have implications for interpreting clinical future studies performed with HIPEC. To our knowledge, this is the first time this pharmacologic information regarding intraperitoneal chemotherapy access to mesenteric lymph nodes has been reported.

## Highlights

• Hyperthermic intraperitoneal chemotherapy (HIPEC) is being used increasingly for peritoneal metastases.
• The distribution of intraperitoneal chemotherapy to target tissues remains poorly described.
• Access of intraperitoneal doxorubicin to mesenteric lymph nodes was available for determination by HPLC.
• Data suggests direct access of intraperitoneal doxorubicin to mesenteric lymph nodes in high concentration.
• High concentrations of intraperitoneal chemotherapy to mesenteric lymph nodes suggests expanded treatment options.

## 1 Introduction

One of the major issues in the administration of cancer chemotherapy concerns the access of cytotoxic concentrations of drug to target tissues. Although this information is available in animal models it is difficult, usually impossible in patients. With regular access to mass spectroscopy and/or high pressure liquid chromatography (HPLC), the concentrations in biological fluids and tissues of cancer chemotherapy can be measured with high accuracy. An unusual clinical situation that allows sampling of blood, peritoneal fluid, and abdominal/pelvic tissues is hyperthermic intraperitoneal chemotherapy (HIPEC) administered in the operating room using the open method [1]. Our efforts have resulted in the routine measurements of chemotherapy access to biological fluids and tissues for a large number of cancer chemotherapy agents. The samples of blood, peritoneal fluid and tissue were possible throughout an open HIPEC procedure in the absence of any invasive pharmacologic monitoring [[2], [3], [4], [5], [6], [7], [8]]. Occasionally, the removal of unusual tissues for pharmacologic monitoring added to the accurate prediction of response to chemotherapy treatments. In the two patients presented in these case reports, resection of mesenteric lymph nodes to determine suspected lymph node invasion allowed the assessment of cancer chemotherapy in these tissues. This information had not been previously available in humans. The limited data on these two patients allow additional hypotheses to be generated regarding cancer chemotherapy distribution after intraperitoneal drug administration.

## 2 Methods

Data on these 2 patients was prospectively recorded and then retrospectively reviewed at an academic institution. All information was from a single academic institution and the cases were consecutive. This research work has been reported in line with the PROCESS criteria [9]. This study was registered as a case series on the www.researchregistry.com website with UIN 4418. Pharmacologic monitoring is a routine part of HIPEC treatment at this institution.

Lymph nodes were resected and submitted for histopathologic examination during the open HIPEC procedure. An approximate 2 mm in diameter portion of the lymph node or tumor nodule sent to pathology was touched to a dry gauze to remove excess chemotherapy solution. The chemotherapy solution that filled the abdominal and pelvic space was manually distributed using an open technique [1]. The dose of doxorubicin was 15 mg/m^2^ in 1.5% dextrose peritoneal dialysis solution [10]. Volume of chemotherapy solution was 1.5 L/m^2^. The preparation of the tissue, peritoneal fluid and plasma samples and HPLC assays were performed by a biochemist of over 30 years experience. Tissues were processed for analysis of doxorubicin concentration as previously described by Sugarbaker et al. [6]. An approximate 100 mg tissue sample was first finely morcellated then accurately weighed and homogenized in 500 μL methanol using a variable speed micro tissue homogenizer (Tissue Tearor, Biospecs Inc., Clearmont, FL, USA). The resulting homogenate containing extracted doxorubicin was filtered through a 0.45 μm syringe filter before the solute was injected directly into the HPLC system. A similar technique was used for the preparation of peritoneal fluid samples after dilution with the appropriate volume of methanol. For plasma samples, 750 μL of methanol was added to 250 μL of plasma. The mixture was then carefully filtered through a 0.45 μm syringe filter before injecting into the HPLC system. Doxorubicin concentrations were determined over time by HPLC. Plasma, peritoneal fluid, and lymph node tissue were available in two patients from which to determine doxorubicin concentrations. Small tumor nodules were available in one of these patients.

HPLC assays were performed using a modified version of the procedure described by Sugarbaker et al. [6] for assessment of doxorubicin levels in peritoneal fluid, plasma and tissue samples. The HPLC system consisted of a Shimadzu LC7A instrument equipped with an SPD-6AV (UV-VIS) detector set at 295 nm and a C-R8a ‘Chromatopac’ data processor (Shimadzu Instruments, Columbia, MD, USA). A reversed C18 column (Varian Associates, Walnut Creek, CA, USA) was used for chromatographic separation. The mobile phase consisted of 28% acetonitrile in 0.1% orthophosphoric acid with 0.1% triethylamine. The flow rate was 1.2 ml/minute and samples were injected through a 50 μL injector loop.

## 3 Patient 1

A 49 year old woman complained of abdominal pain in November of 2015 with diagnosis of large uterine fibroid, a myomectomy from within the uterus was performed. No malignancy was associated with the specimens recovered. In December of 2016, the abdominal pain persisted and a mass was palpable on physical examination in the mid-abdomen on the right. Colonoscopy was performed and biopsy showed a moderately differentiated adenocarcinoma of the caecum.

CT was performed in December of 2016 and the primary right colon malignancy along with bilateral ovarian metastases were imaged. Percutaneous biopsy of the right ovarian mass showed well differentiated adenocarcinoma consistent with a colonic primary. From December of 2016 through December of 2017, the patient was maintained on chemotherapy. Initially, she was treated with FOLFOX. After four cycles, because of neuropathy, the oxaliplatin was stopped. The patient was maintained on 5-fluorouracil and bevacizumab.

In December of 2017, a CT documented marked regression of the primary tumor. It was not visible by CT. However, multiple lymph nodes within the mesentery of the distal small bowel were enlarged. Also by CT a pelvic mass showed that the right ovary had increased in size to 15 cm in greatest diameter despite the fact that the primary caecal malignancy was no longer visible by CT.

**Fig. 1:**
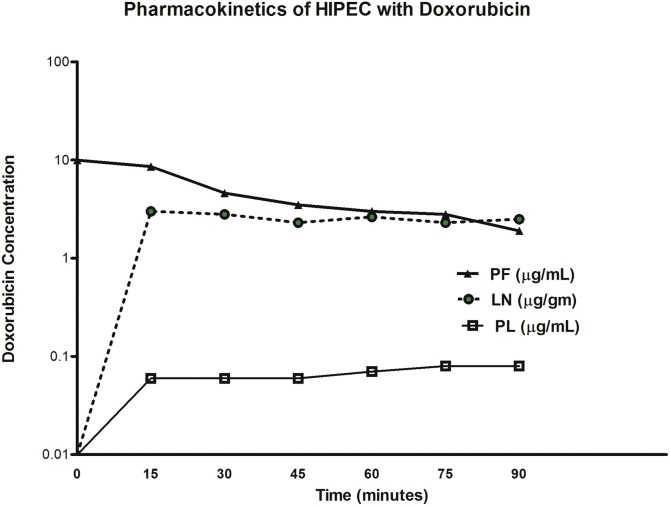
In this patient with caecal adenocarcinoma, enlarged lymph nodes in the small bowel mesentery were resected and subjected to histologic and pharmacologic study. Drug concentrations in peritoneal fluid (PF) and plasma (PL) were also determined. Doxorubicin concentration in lymph nodes (LN) was markedly increased over that in plasma and was very similar to the intraperitoneal drug concentration between 60–90 min.

Over approximately one month the patient became rapidly more symptomatic with abdominal distention from ascites and an expanding right ovarian mass. On February 1, 2018 the patient underwent cytoreductive surgery and HIPEC in a specialized center for management of peritoneal metastases. At the time of surgery she underwent a greater omentectomy, hysterectomy, bilateral salpingo-oophorectomy, complete pelvic peritonectomy, right colon resection, and small bowel resection. She received HIPEC with mitomycin C and doxorubicin with systemic fluorouracil. In order to determine if the multiple enlarged lymph nodes within the small bowel mesentery were involved by cancer, individual lymph nodes were removed and subjected to histopathologic study by permanent sections at a later time. A portion of each of these lymph nodes was sent for pharmacologic analysis for doxorubicin content. Fig. 1 shows the pharmacokinetics of intraperitoneal doxorubicin in peritoneal fluid, mesenteric lymph nodes, and plasma. Clearly, the lymph nodes have taken up large amounts of doxorubicin nearly equal to that which was seen within the peritoneal fluid. The increased amounts of doxorubicin within lymph nodes as compared to the plasma can be measured by the area under the curve ratio. The ratio of lymph nodal tissue concentration times time to plasma concentration times time was 40. The area under the curve ratio (AUC ratio) of peritoneal fluid to plasma was 80.

After the HIPEC chemotherapy and biopsies, an end-to-side coloenteric anastomosis was performed. The patient required a 3-week hospitalization and was discharged eating well and having normal bladder and bowel function. Pathology showed cancer in the right ovary and small amounts of cancer remaining at the primary site. No cancer was present in the mesenteric lymph nodes.

## 4 Patient 2

This 35 year old woman began noticing increasing fatigue in 2014. The fatigue and left lower quadrant pain became increasingly severe so that she went an emergency room. On 19 February 2018, a CT scan was performed that showed an infiltrate into the greater omentum and multiple nodules associated with small bowel mesentery. There was no ascites. Masses were present on both the right and left ovaries. On 14 March 2018, she was taken to the operating room where multiple biopsies were taken. Her left ovary and appendix were removed. Pathology showed a mature cystic teratoma and extensive malignant peritoneal mesothelioma. Multiple biopsies from omentum, surface of uterus, peritoneum from the abdominal wall, and peritoneum from the small bowel showed malignant peritoneal mesothelioma.

**Fig. 2:**
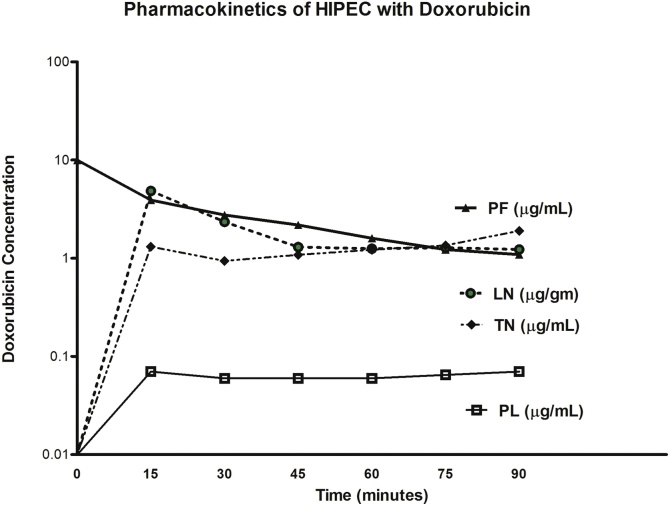
In this patient with malignant peritoneal mesothelioma, doxorubicin concentrations in peritoneal fluid (PF), plasma (PL), mesothelioma tumor nodules (TN) and lymph nodes (LN) within the small bowel mesentery were determined. High concentrations of drug were present within small tumor nodules bathed by the chemotherapy solution. Similar high concentrations of doxorubicin were present in lymph nodes buried within the mesenteric fat of the small bowel.

Repeat CT on 2 May 2018 showed malignant peritoneal mesothelioma infiltrating the omentum and accumulating as a diffuse mass in the pelvis. The small bowel mesentery showed multiple prominent lymph nodes. For definitive treatment of her malignant peritoneal mesothelioma, the patient was taken back to the operating room on 3 May 2018. At that time she had a greater and lesser omentectomy, cholecystectomy, pelvic peritonectomy with hysterectomy and right oophorectomy along with cytoreductive surgery of the small and large bowel surfaces. HIPEC was performed with cisplatin, doxorubicin, and systemic ifosfamide with Mesna (2-Mercaptoethanesulfonic acid sodium). During the HIPEC procedure Mayo scissor dissection removed a layer tumor from large and small bowel surfaces along with large and small bowel mesenteric surfaces [11]. In order to rule out disease within the mesenteric lymph nodes, six of these lymph nodes were harvested during the HIPEC chemotherapy [12]. Portions of the node were sent for histopathologic analysis and other portions were sent for pharmacologic analysis of the intraperitoneal drug, doxorubicin. The results of the doxorubicin within blood, peritoneal fluid, lymph nodes, and tumor are shown in Fig. 2. The area under the curve ratio of lymph node doxorubicin to plasma doxorubicin was 30. The area under the curve ratio of tumor nodules to plasma was 20 and from peritoneal fluid to plasma was 50.

Again in this patient the markedly increased levels of doxorubicin in lymph nodes as compared to plasma were documented.

The patient was hospitalized for 15 days and was discharged eating well. An intraperitoneal port was placed at the time of the cytoreductive surgery and will be used to deliver long-term intraperitoneal pemetrexed with systemic cisplatin. Six cycles of treatment are planned. Pathology of resected specimens showed malignant peritoneal mesothelioma; the lymph nodes were negative for cancer.

## 5 Discussion

In both patients an assessment of doxorubicin concentration in lymph nodes was greatly elevated over that observed in plasma. In both patients after 90 min of hyperthermic intraperitoneal lavage the doxorubicin concentration in mesenteric lymph nodal tissue was near the same magnitude as in peritoneal fluid. In these patients a mechanism to explain the movement of high concentrations of doxorubicin from peritoneal fluid to lymph nodes in the absence of a distribution by the vascular system must be identified.

Prior assessments of chemotherapy concentration in mesenteric lymph nodes in humans after intraperitoneal drug administration are lacking. However, prior animal studies have shown a large uptake of chemotherapy by mesenteric lymph nodes as compared to tissues in the body compartment. Pestieau et al. determined the pharmacokinetics of intraperitoneal pemetrexed in Sprague-Dawley rats [13]. The AUC of peritoneal fluid concentration times time to plasma concentration times time over a 3-hour time period was recorded. The AUC ratio was 40.8. After 3 h of intraperitoneal instillation, the concentration of pemetrexed was statistically significantly increased (p = 0.0036) when compared to mesenteric lymph nodes from rats given the same dose of pemetrexed intravenously.

Soma et al. studied the tissue distribution of paclitaxel after intravenous and intraperitoneal drug administration in white Japanese rabbits [14]. At one-half hour after administration, intravenous drug concentration was higher than intraperitoneal drug concentration in mesenteric lymph nodes. However, at 6 h the paclitaxel concentration in mesenteric lymph nodes after intraperitoneal administration was twice as high as after intravenous administration (p < 0.05). At 24 h the intraperitoneal drug concentration was four times that of intravenous paclitaxel (p < 0.05).

Of course, these limited observations in only two patients with a single chemotherapy agent need to be confirmed in pharmacologic studies in a large number of patients. Other drugs pharmacologically appropriate for intraperitoneal chemotherapy administration must be studied to determine the relevance of these limited data. Nevertheless, some speculation regarding the access of such large amounts of doxorubicin to mesenteric lymph nodes is warranted.

It is highly unlikely that direct diffusion of doxorubicin through peritoneum and mesenteric fat caused these high concentrations within lymph nodes. Simple diffusion is likely to be the mechanism for high doxorubicin concentration in small tumor nodules shown in Fig. 2. Ozols et al. determined that the penetration of this drug into tissues was limited to a few cell layers [15]. Jacquet et al. showed that the limited doxorubicin penetration was increased by heat [16]. However, direct diffusion through several millimeters of normal tissue is extremely unlikely.

Access of the intraperitoneal doxorubicin to mesenteric lymph nodes through the bloodstream is also highly unlikely. Plasma concentrations throughout the 90-minute HIPEC are approximately 30–40 times less than the lymph node concentrations. The most likely access to the mesenteric lymph nodes is through the subperitoneal lymphatics. This is a lymphatic network that is well developed on both parietal and visceral peritoneum [17]. Although chemotherapy entrance into the lymphatic structures on the peritoneal surface and then into mesenteric lymph nodes has not been previously demonstrated, it seems to best explain doxorubicin present in high concentration in lymph nodes.

Prior to these observations, chemotherapy clearance from the peritoneal space was considered to occur largely by way of the portal blood. This was suggested by Speyer et al. in their studies in humans with 5-fluorouracil [18]. They reported elevated portal 5-fluorouracil concentrations as compared to concentrations in peripheral blood and postulated that large quantities of this drug were metabolized as a single pass through the liver. The pharmacologic studies of 5-fluorouracil in mesenteric lymph nodes would have been most interesting but no observations were reported.

It is possible that these observations regarding a subperitoneal lymphatic network have clinical implications. In this study the intraoperative chemotherapy solution was delivered by the open technique using manual distribution. An alternative would be a closed abdomen technique [19]. No differences in the pharmacology of open versus closed HIPEC procedures have been reported. However, when intraperitoneal chemotherapy is administered by repeated cycles of treatment over many months, the free access of the chemotherapy solution to all peritoneal surfaces would be limited by adhesions. One possible explanation for the success of intraperitoneal chemotherapy long-term despite very limited distribution within the abdomen and pelvis may be another mechanism for chemotherapy distribution in addition to the free peritoneal space. One candidate for this is the subperitoneal lymphatic network. In our two patients a pharmacologic study of the mesenteric lymph nodes revealed very high levels of doxorubicin nearly as high as the chemotherapy concentrations within the peritoneal space. These data suggest a large uptake of intraperitoneal doxorubicin chemotherapy into lymphatic channels and into mesenteric lymph nodes. This rapid diffusion of cancer chemotherapy throughout the visceral peritoneum may help to explain the large abdominal and pelvic response to intraperitoneal chemotherapy despite its inadequate distribution after prior extensive surgical dissection [20].

## 6 Conclusions

In these two patients the access of doxorubicin chemotherapy to mesenteric lymph nodes from the chemotherapy solution within the peritoneal space was determined. Nearly the same concentration of chemotherapy was present within the lymph nodes as within peritoneal fluid. The plasma concentrations were much lower suggesting that chemotherapy access to the mesenteric lymph nodes was through subperitoneal lymphatic channels.

## Conflicts of interest

The authors has no conflicts of interest to declare.

## Funding

Data management and secretarial support provided by Foundation for Applied Research in Gastrointestinal Oncology.

## Ethical approval

MedStar Health Institutional Review Board has determined that a case report of less than three (3) patients does not meet the DHHS definition of research (45 CFR 46.102(d)(pre-2018)/45 CFR 46.102(l)(1/19/2017)) or the FDA definition of clinical investigation (21 CFR 46.102(c)) and therefore are not subject to IRB review requirements and do not require IRB approval.

This case series is of 2 patients.

## Consent

Written and signed consent was obtained from the patients.

## Author contribution

Paul H. Sugarbaker, MD and O. Anthony Stuart: study concept or design, data collection, data analysis or interpretation, writing the paper

## Registration of research

This study was registered as a case series on the www.researchregistry.com website with UIN 4418.

## Guarantor

Paul H. Sugarbaker, MD

## Provenance and peer review

Not commissioned, externally peer-reviewed
